# Individual differences in language during childhood predict well-being in adolescence

**DOI:** 10.1093/chidev/aacaf057

**Published:** 2026-02-26

**Authors:** J Bruce Tomblin, Isaac T Petersen, Melissa S Hill, Kristi I Hendrickson

**Affiliations:** Department of Communication Sciences and Disorders, University of Iowa, Iowa City, IA, United States; Department of Psychological & Brain Sciences, University of Iowa, Iowa City, IA, United States; Department of Communication Sciences and Disorders, University of Iowa, Iowa City, IA, United States; Department of Communication Sciences and Disorders, University of Iowa, Iowa City, IA, United States

**Keywords:** well-being, language ability, adolescence

## Abstract

This study examined whether early language ability influences adolescents’ well-being, using longitudinal data from 502 children (223 females, 279 males; 86% White, 12% Black, 1% Hispanic, and 1% Asian). Measures of oral language, performance IQ, and socioeconomic status were obtained during elementary grades. At ages 16–17, psychological well-being was assessed using the *Perceived Competence Scale* (comprising scholastic ability, self-esteem, and friends factors) and *Satisfaction with Life* scale. Structural equation modeling showed language ability significantly predicted later scholastic ability and friendship. Scholastic ability mediated the association between language ability and satisfaction with life. Socioeconomic status moderated associations between language and scholastic ability. Findings support that individual differences in oral language ability affect adolescents’ academic and social self-competence perceptions and life satisfaction.

Oral language acquisition and use is widely regarded as a unique and universal human ability that allows humans to influence each other's thoughts and behaviors and transmit culture across generations. In this study we focus on the ability to comprehend and produce spoken utterances. The functions served by language are recognized as essential to the evolutionary success of humans (Laland & Seed, 2021) and are central to human social function. Although language is a universal trait among humans, individuals, even within the same community, vary with regard to their facility in understanding and producing utterances (Bates et al., 1995). Researchers have shown that vast individual differences in language ability emerge in early childhood and persist throughout the school-age years (Tomblin et al., 2014). During the school years, oral language skills are well known to enable and influence a wide array of educational achievements (see for instance: Spencer et al., 2017) and support social and behavioral interactions (Petersen et al., 2013; Snow, 2014). We can thus hypothesize that individual differences in oral language will shape how children think and feel about themselves, thereby influencing their subjective or personal well-being. The current study asks whether individual differences in children's language ability across the ability spectrum during their school years contribute to their sense of well-being as they approach adulthood.

Several terms are often employed to refer to some aspect of the quality of a person's general life status. The terms, quality of life, standard of living, satisfaction with life (SWL), and well-being share a similar semantic space and the meanings vary. We will follow what appears to be a common scheme shown in Figure 1. The common space for these terms could be the notion of quality of life. Within quality of life, most accounts (Boelhouwer & Noll, 2014; Cummins, 2000) distinguish between objective and subjective ways of characterizing the quality-of-life status. Objective measures often reflect personal resources such as income, education, and occupation often called socioeconomic status (SES). In contrast with objective SES status is personal well-being. In this case, the source of quality-of-life information is the individual's report and judgment of themselves. Martela and Sheldon (2019) and, more recently, Ryff et al. (2021) argue that well-being is both “feeling good” and “doing well.” In the first case (feeling good), the emphasis is on feelings of pleasure, happiness, and satisfaction and thus reflects an emotional or hedonic state (Pavot & Diener, 2008). This form of well-being has often been termed subjective well-being. The second form of well-being (doing well) focuses on an individual's functioning within their current environment, are often described in such terms as flourishing, life purpose, and personal development. This version of well-being has been termed eudaimonic well-being or psychological well-being (Keyes et al., 2002). Finally, Das and colleagues (2020) have noted that measures of SWL are reflective of the individual's personal values, whereas the measures of psychological well-being ask about how the person is doing regarding culturally valued areas of function and are thus normative. In this study, we examined the links between language and measures of both subjective well-being (hedonic) and psychological well-being (eudaimonic).

**Figure 1:**
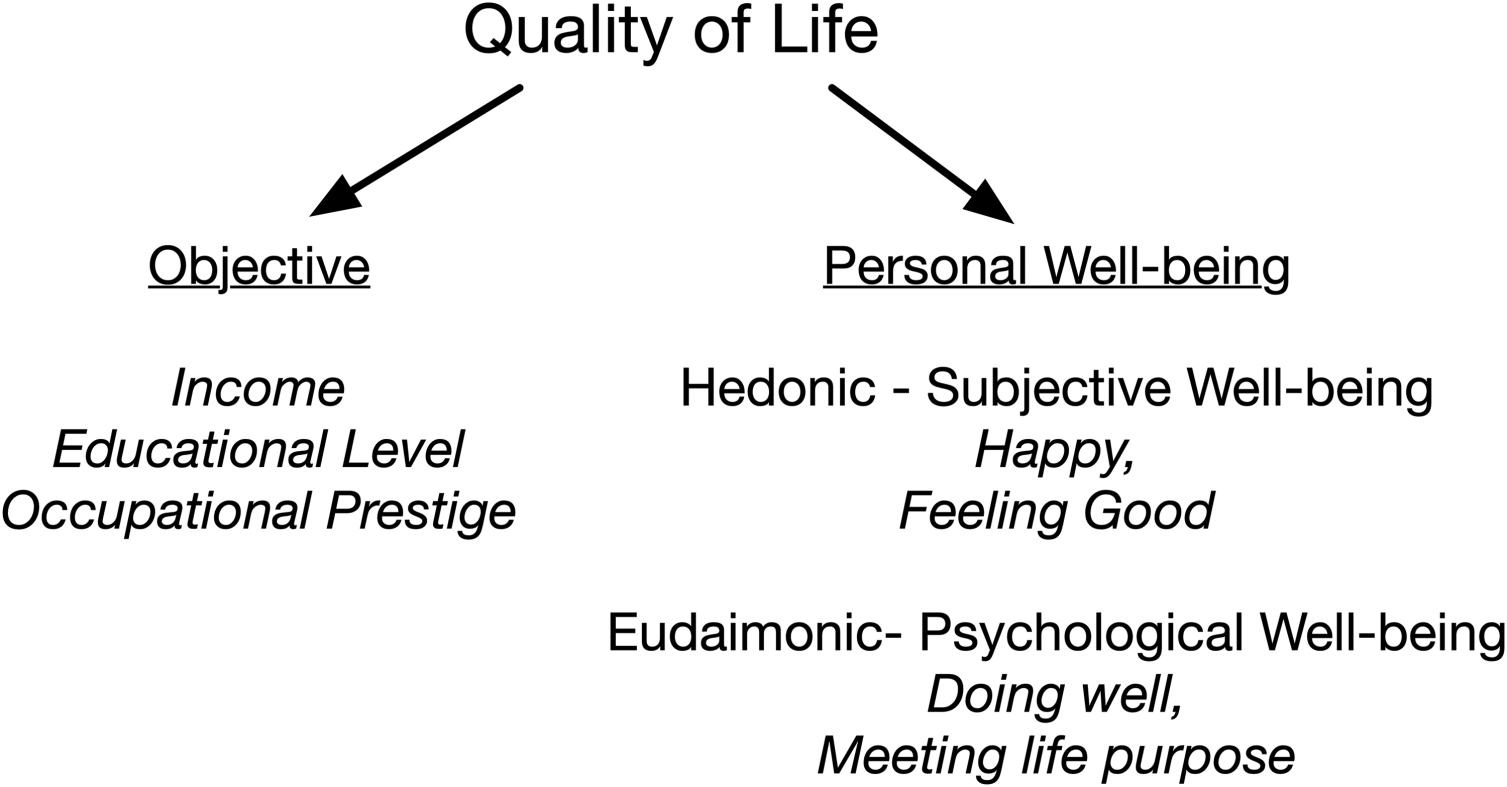
A conceptual framework of well-being and quality-of-life constructs examined in this study.

Researchers have developed several measurement scales to quantify either one or both forms of well-being. Empirical evidence using factor analysis supports the distinction between subjective well-being and psychological well-being (Keyes et al., 2002; McGregor & Little, 1998). However, several studies have found that scales measuring these two aspects of well-being are often highly correlated. A recent bi-factor analysis revealed a nested structure, with each aspect of well-being representing a separate dimension within a more general common factor (Khanna et al., 2024). Although subjective well-being and psychological well-being are associated, it is not clear whether one drives the other. Keyes et al. (2002) noted that research is needed to examine how psychological well-being and subjective well-being influence each other and that longitudinal data are needed to address this issue. The data from this longitudinal study will afford an opportunity to do this.

Despite language's fundamental role in human development, research directly examining the relationship between language ability and well-being remains surprisingly limited. Riad and coll­eagues (2023) obtained a self-report of well-being in five-year-old children using a Swedish version of the How I Feel About My School questionnaire. This measure asks children seven questions regarding how they feel (Happy, OK, and Sad) in different settings at school and, thus, can be viewed as a measure of subjective well-being. These subjective ratings and the child's vocabulary and narrative performance were not correlated. Nikolaev and McGee (2016) studied a large sample of adults and reported a weak positive association between an index of relative standing derived from a 10-word vocabulary test and subjective well-being measured by a single item: “Taken all together, how would you say things are these days? Would you say that you are very happy, pretty happy, or not too happy?”.

A larger number of studies have contrasted measures of well-­being between children with low language ability such as developmental language disorder (DLD) and children with more typical ability. Lyons and Roulstone (2018) reported a qualitative study of interviews with 11 children with speech and language disorders. Concerns over academic and social relationships were often voiced by these children. Three studies have examined hedonic well-being judgments among children and young adults with and without DLD (Conti-Ramsden et al., 2016; Gough Kenyon et al., 2021; Records et al., 1992). The children with DLD and typical controls had similar levels of subjective well-being ratings. Thus, language, at best, has a small effect, if any at all, on well-being. However, it is important to note that the limited extant work has focused exclusively on subjective well-being.

Evidence of an association between language and well-being may also be found from indirect evidence. Language ability in childhood is well correlated with school achievement, SES status of the home, and intelligence. Therefore, if language ability plays a role in the well-being of children and adolescents, it is likely to be associated with these factors as well. Two recent meta-­analyses have examined the association between educational performance, intelligence, and well-being in children and adolescents. Based on 41 studies, Bucker and colleagues (2018) found that the correlation between academic achievement and subjective well-being was small. Similarly, Kaya and Erdem (2021), based on the data from 81 studies, found the overall mean correlation between students’ well-being and academic achievement was also small (average *r* = 0.17). Thus, among children and adolescents, well-being is associated with better school performance; however, the effect size is small.

The association between well-being and intelligence has been extensively examined. Veenhoven and Choi (2012) summarized the literature on intelligence and happiness (subjective well-­being), finding little evidence of an association between these. Subsequently, Ali et al. (2013) reported a small effect of intelligence on subjective well-being. Wigtil and Henriques (2015) used the SAT in college students to measure intelligence and found a larger effect when well-being was a measure of psychological well-being. Also, Dimitrijević et al. (2018) found that intelligence was associated with psychological well-being in adults; however, SES status accounted for all this association. More recently, Ma and Chen (2024) found no evidence of an association between several measures of intelligence and SWL. Thus, intelligence is, at best, weakly related to subjective well-being in adults; however, it may be more impactful on psychological well-being.

We began by asserting that language is a highly important function among humans and even more so in developed countries. As such, we might expect that individual differences in language abilities during childhood would directly or indirectly influence children's quality of life, including their well-being; however, very little empirical data explicitly speaks to this conclusion. Even the research examining covariates of language such as intelligence and academic performance provides, at best, a very small effect on the role of language ability in well-being. In this study, we employed data from an 11-year longitudinal study of a large epidemiologically sampled cohort that we followed from kindergarten through the 11th grade. We obtained extensive subjective well-being measures that reflected hedonic or eudemonic aspects of well-being during the 10th and 11th grades. Language measures were obtained at four time points during elementary school and thus preceded the well-being measures. Two potential confounders—nonverbal intelligence (performance IQ, PIQ) and home SES status—were also measured during the elementary grades. Controlling for performance intelligence increased the likelihood that any association of language with well-being was specific to language rather than domain general cognition. Also, because our language measures were obtained in a standardized environment similar to that used for PIQ we increased our control of this confounder. Socioeconomic status is also well known to be associated with both language (Hart & Risley, 1995) and well-being (Tan et al., 2020) and thus could confound interpretation of language—well-being associations. Based upon this reasoning, this study used structural equation modeling (SEM) to test the direct effects of early individual differences in language on subsequent subjective well-being while controlling for PIQ and SES status.

A second analysis was performed to examine whether cultural factors reflected by SES might moderate the association of language with well-being. We noted earlier that subjective well-being is inherently normative, and SES status is reflective of cultural differences. Thus, SES status is associated with differences in individuals’ patterns of thought and behavior (Côté, 2011; Kraus et al., 2011). Accordingly, individuals from different SES backgrounds may interpret and respond to well-being measures in systematically different ways. This suggests that SES status could function as a moderator of the strength of the association between language ability and well-being.

A third analysis was motivated by Keyes et al. (2002) observation that eudaimonic (psychological well-being) and hedonic (subjective well-being) might interact. In this study the eudaimonic measures were obtained a year before the hedonic measures. Therefore, we employed a mediation model to examine whether there were indirect relationships between language and SWL that depended upon the earlier eudaimonic states.

## Method

### Participants

The data used in this study were from 502 (223 females, 279 males) high school students in 10th and 11th grades during the years 2003–05. These students were members of a longitudinal cohort of 604 children initially enrolled as kindergarteners (see Nippold & Tomblin, 2014 for a full description of the study methods). This research was conducted under the supervision and approval of the University of Iowa Human Subjects Committee. The participants were informed and consented to participate and data collection.

The racial makeup of the participants was 86% White, 12% Black, 1% Hispanic, and 1% Asian. During the fourth grade assessment, we obtained parental educational level and household income. The median educational level of the mothers was 13 years (1st quartile 12), and of the fathers was 12 years (1st quartile 12). Participants reported their household income in $10,000 increments, ranging from less than $20,000 to above $90,000. The median income for these participants fell in the $40–$50 k interval, with the first quartile in the $20–$30 k interval. The national median income was $42,000 at this time.

We selected the children in this study from a large cross-sectional sample of kindergarten children living in Iowa and western Illinois who participated in an epidemiologic study (Tomblin et al., 1997). The cross-sectional epidemiologic study examined with the prevalence and risk factors of specific language impairment (now DLD). It began with a population sample of nearly 7,000 children in kindergarten who were given a short oral language test. Based on this initial screening a case-control sample of 1,929 with an oversampling of children with poor oral language was selected and administered a more extensive battery of language, nonverbal IQ, and prereading tests. Subsequently, in grade 2, 604 (244 DLD, 315, typically developing) children were recruited for the longitudinal study using a case-control design. This case-control sample comprised all monolingual English-speaking children except for those with known hearing loss, severe visual impairment, autism, or intellectual disability. Due to its focus on DLD, we oversampled children with poor language abilities. Because the case-control sample was systematically derived from a population sample, we were able to calculate inverse probability weights representing each child's probability of selection from the initial 7,000 screening participants (Tomblin, 2014; Tomblin et al., 1996). These weights were applied in all analyses in this study to correct for the oversampling bias.

### Measures

A set of measures obtained across the 11-year study period was used to derive latent measures of language, nonverbal IQ, SES status, psychological well-being and SWL. The measures of psychological well-being were initially examined via exploratory factor analysis to determine the dimensionality of the items in this scale. The resulting subscales of psychological well-being were then incorporated into a measurement model using confirmatory factor analysis along with the measures of SWL, language, PIQ, and SES. Table 1 outlines the time points each of the measures was obtained in the longitudinal design.

**Table 1:** School grades at which the measures were obtained.

Construct	Kindergarten	Second	Fourth	Eighth	Tenth	Eleventh
**Language**	✓	✓	✓	✓		
**Performance IQ**	✓	✓		✓		
**SES**			✓			
**Psychological Well-being**					✓	
**Satisfaction with Life**						✓

#### Language

Language comprehension and production were assessed four times between kindergarten and eighth grade. The language assessment battery is described in Appendix A. The measures were common standardized measures of listening and speaking designed for children in the school years. The dimensionality of these measures was examined using an exploratory factor analysis (Tomblin & Zhang, 2006). Much of the variance in these measures was attributable to a single common factor. Thus, language at each assessment wave was summarized as an average *z*-score composite measure of relative standing. These *z*-scores represented local (sample) norms based on the inverse probability weighting value described earlier. These four language measures across development were also highly correlated (Mean *r* = 0.82, min = 0.76 max = 0.87) and thus were used as indicators of a single latent language trait in a measurement model extending across the elementary school years.

### Performance intelligence quotient

We included a measure of PIQ in standard score units in the structural model to account for nonlinguistic cognitive skills that could potentially be confounded with language ability. Performance IQ was measured in kindergarten using the *Block Design* and *Picture Completing* subscales of the *Wechsler Preschool and Primary Scale of Intelligence-Revised* (Wechsler, 1989) and in second, and eighth grades using the *Wechsler Intelligence Scale for Children-III Performance Scale* (Wechsler, 1997) consisting of: *Picture Arrangement, Picture Completion, Block Design, Object Assembly*, and *Coding*. These composite measures were well correlated across the waves of testing (Mean *r* = 0.69, min = 0.66 max = 0.73). We used these measures as three indicators of the latent factor of PIQ across ages.

### SES status

In fourth grade, the mother and father provided information concerning the highest grade they attended. They also reported the household income in increments of $10k from <$20k to >$100k. These measures served as manifest indicators of household SES status in the measurement model and within structural models as a possible confounder of language and a possible moderator of language effects on well-being.

### Measures of well-being

We used two scales that reflected different aspects of well-being. One measured the participant's self-perception of competence during the 10th-grade assessment wave. This scale addressed important aspects of adolescent functioning, which we categorized as forms of eudemonic or psychological well-being. Although this scale was not explicitly a scale of psychological well-being, Ryan and Deci (2001, p. 156) noted that “feeling competent and confident with respect to valued goals is associated with enhanced well-being.” Also, in 11th grade, we administered the *Satisfaction with Life* Scale (Diener et al., 1985). This scale is generally regarded as measuring a hedonic form of well-being.

*Psychological well-being*. This questionnaire was modeled after the *Perceived Competence Scale for Children* (Harter, 1982), which measures cognitive, social, and physical competence and self-worth. The items are consistent within the construct of eudaimonic well-being, and researchers have viewed it as a measure of well-being (Mann et al., 2004). This scale employs a “structured alternatives” scaling method designed to control for socially desirable responses by asking the subjects to compare themselves to examples. As shown in Table 2, the students are given two contrasting statements that can be applied to them. They are to select which one most applies to them and then select a second statement representing the degree to which this applies. The scores on these are then assigned with the highest value representing the most desirable level of competence. For some items, the highest was displayed on the left, and for others, it was on the right.

**Table 2:** Example item using structured alternative scaling.

Some students like the kind of person they are		Other students wish they were different	
Really true for me	Sort of true for me	Sort of true for me	Really true for me
4	3	2	1

Sixteen items (see Appendix B) concerning various aspects of personal self-worth were drawn from the *Self-Perception Profile for Adolescents* (Harter, 1988). Because we were interested in associating language abilities with these constructs, some items were revised to simplify the language. Also, these items were presented auditorily via computer to reduce the influence of reading abilities. Harter has shown that there are multiple latent dimensions to self-perception of competence. Thus, we examined these data to determine the best measurement structure to be used in the SEM. We performed an exploratory factor analysis using maximum likelihood extraction with promax rotation on inverse probability weighted Spearman correlations, implemented via the fa function from the psych package (version 2.14.22) in R (version 4.4.3). We initially examined factor models ranging from 1 to 5 factors using, evaluating fit using root mean square approximation (RMSEA), Bayesian information criterion (BIC), and Tucker-Lewis index (TLI) indices. RMSEA indicated acceptable model fit for solutions with 2 or more factors (range: 0.074–0.036). BIC reached its minimum at 4 factors (−227.6), suggesting optimal parsimony at this level. While TLI reached acceptable levels (.92) at 5 factors, the four-factor solution was acceptable (0.88). The final 4-factor model is presented in Table 3.

**Table 3:** Personal self-worth indicator loadings (=>0.30) on four latent variables from an exploratory factor analysis.

Scale item	Scholastic ability	Self-esteem	Social-physical ability	Friendships	Communality
**Smart**	0.85				0.56
**Confident with school**	0.73				0.5
**Good school performance**	0.70				0.44
**Understand teacher**	0.57				0.43
**Bright**	0.54				0.32
**Creative**					0.06
**Like appearance**		0.71			0.58
**Like self**		0.65			0.48
**Self-satisfied**		0.52			0.42
**Positive parent relationship**		0.47			0.30
**Have romantic relationship**			0.54		0.37
**Good dancer**			0.41		0.17
**Athletic**			0.33		0.18
**Comfortable talking**			0.31		0.29
**Not lonely**				0.65	0.42
**Have close friend**				0.47	0.28

This factor structure was very similar to those reported by Harter (2012) where she reported four factors of: scholastic, social, athletic, and appearance. We assigned items to factors when loadings were greater than.30. We interpreted Factor 1 (smart, confident with school, bright, good school performance, understand teacher) as reflecting scholastic ability. Factor 2 (like appearance, like self, self-satisfied, and positive parent relationship) can be viewed as reflecting self- esteem. Factor 3 (have romantic relationship, good dancer, athletic, comfortable talking) as reflecting social-physical ability Factor 4 (not lonely have close friend) was considered as reflecting friendships and companionship. Creative was not loaded on any factor and thus was dropped from the subsequent analyses.

*Satisfaction with Life*. The SWL scale (Diener et al., 1985) is generally regarded as a measure of hedonic well-being. We administered this scale when the participants were in the 11th grade. This scale consists of five items (see Table 4). Each statement is rated from 1 (*strongly disagree*) to 7 (*strongly agree*). The first four items deal with the person's current state, whereas the fifth asks about the respondent's long-term view of life. We used the first four items as manifest variables of a single latent variable of SWL, as Diener et al. (1985) has concluded the fifth item functions as a global measure and is redundant.

**Table 4:** Satisfaction with life scale items (Diener et al., 1985).

Indicator	Scale items
**SWL-1**	In most ways my life is close to my ideal.
**SWL-2**	The conditions of my life are excellent.
**SWL-3**	I am satisfied with my life.
**SWL-4**	So far, I have gotten the important things I want in life.
**SWL-5**	If I could live my life over, I would change almost nothing.

#### Measurement model of latent variables

*Manifest measures*. The primary question of this study concerned the association between language ability during elementary school years and eudemonic (psychological well-being) and hedonic (SWL) well-being in later adolescence. Twenty-nine manifest variables were hypothesized to be the product of eight latent variables (language ability, PIQ, SES status, scholastic ability, social-physical ability, self-esteem, friendship, and SWL). Appendix C provides descriptive statistics for each variable. For all variables, except those from the SWL scale, 502 observations were available. This scale was administered one year later than the Psychological Well-being measures, and 448 responses were obtained at that time. The overall rate of missing data was 1.8% and 420 of the 502 cases were complete (83.67%). Due to missing data, the pairwise deletion method was employed in the subsequent analyses given the use of a WLSMV estimator to handle ordinal data. The means of the PIQ measures were weighted standard scores (mean = 100, *SD* = 15) based on national test norms, language measures were weighted *z*-scores (mean = 0, *SD* = 1). Indices of skewness and kurtosis showed that the language, PIQ, and SES status measures supported treating these as continuous scales. The indices of well-being rating were on ordinal scales, whereas the language and PIQ were continuous. Correlations among these indicators are shown in Appendix D. Spearman correlation methods were used between the ordinal measures and between ordinal and continuous indicators. Pearson correlations were used among the continuous variables.

*Confirmatory factor analysis of measurement model*. As noted above, this study was primarily concerned with the associations among eight latent constructs. To test this model, we used a confirmatory factor analysis implemented within the lavaan package of R (Rosseel, 2012). We used DWLS estimation with mean and variance adjusted (WLSMV) corrections for categorical indicators and sampling weights, with and χ²tests based on the Satorra–Bentler scaled and shifted χ² test statistic. Throughout, we report scaled fit indices using the Satorra–Bentler correction, which provides more accurate fit assessment when using the WLSMV estimator with ordinal indicators. Standard fit indices assume multivariate normality and continuous variables, making scaled indices more appropriate for our mixed continuous-ordinal measurement model. These adjustments acknowledged the ordinal nature of some of the well-being indicators. Pairwise deletion was used to handle missing data, ensuring that all available data points were used in the analysis. We based the confirmatory factor analysis model on theoretical considerations and in the case of the psychological well-being scales we primarily used the four-factor solution in the EFA (scholastic ability, self-esteem, physical-social competence and friendships). This solution resulted in one factor (friends) having only two indicators which did not allow the CFA to converge. In the three factor EFA, comfortable talking loaded on friends. It is also theoretically consistent with engagement with friends and therefore we moved the comfortable talking indicator to the friends factor which then provided three indicators. The four latent variables were then combined with the four addition latent variables (SWL, SES, PIQ, language) to form a preliminary measurement model. Scaled fit indicators showed the model fit the data well (χ²(349, *N* = 502) = 580.95, *p* = <.001; CFI = 0.943; TLI = 0.934; RMSEA) = 0.036; SRMR = 0.056.

**Figure 2:**
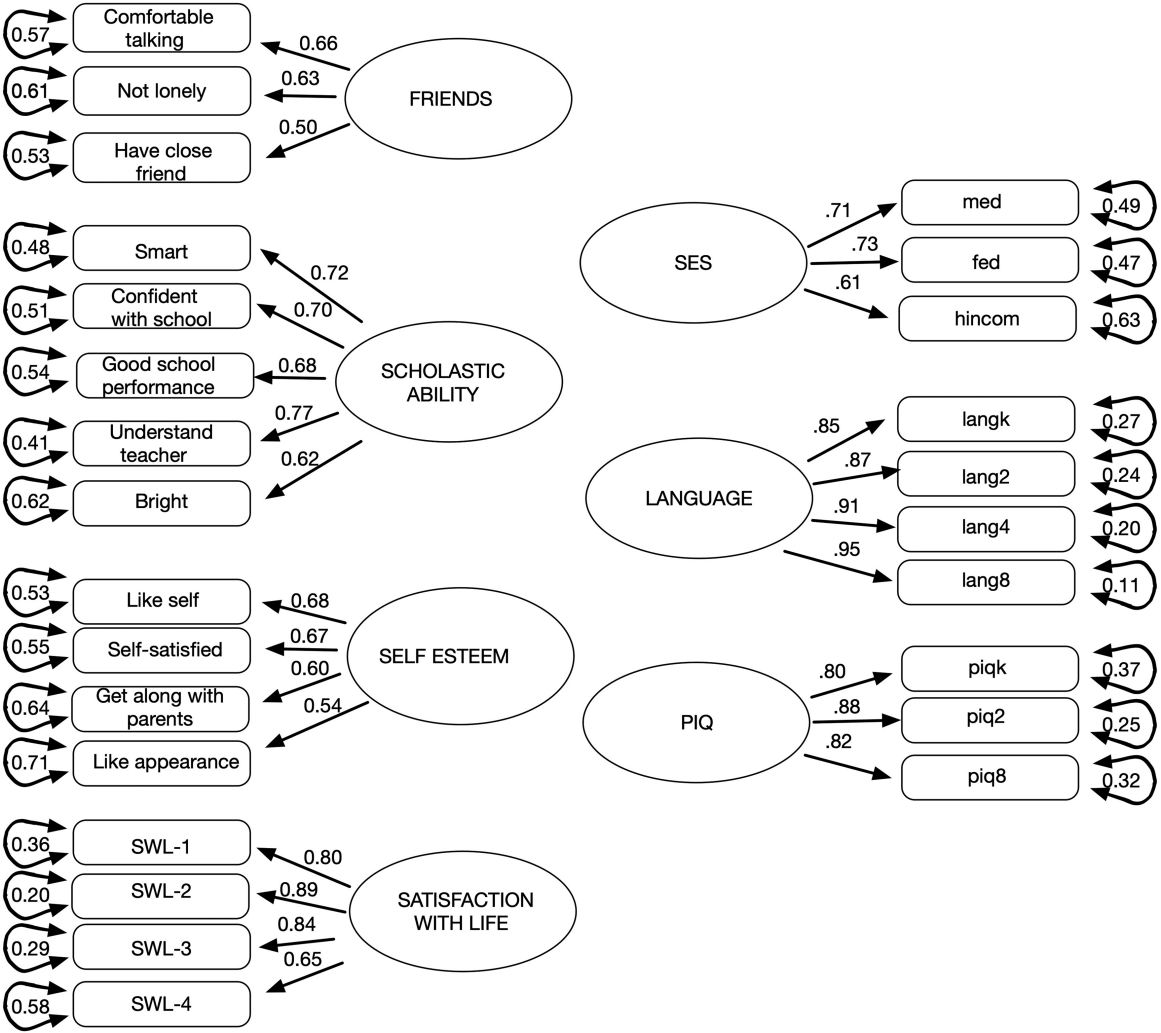
7 factor measurement model used in all SEMs.

Table E1 in Appendix E provides the coefficient loadings of each indicator variable on its respective latent variable, the composite reliability of the latent variables, and the average variance extracted by the latent variable. As shown in Table E1, the latent variable physical-social competence had low indicator loadings on two of the three indicators and had inadequate composite reliability and average variance extracted. Thus, this latent variable was removed from the measurement model. The subsequent 7 factor model well (χ²(278, *N* = 502) = 467.864, *p* = <.001; CFI = 0.952; TLI = 0.943; RMSEA = 0.037; SRMR = 0.052. Figure 2 provides the indicators loading on the final 7 factors and the latent variable correlations for this model. This model served as the measurement model in the SEM described in the results section.

### Structural equation models

This study represents a primarily exploratory investigation. While grounded in theoretical frameworks linking language development to social-emotional outcomes, the specific pathways examined between early oral language ability and adolescent well-being are not well known with limited prior empirical guidance. The structural equation models were developed to test theoretically-motivated associations. Thus, while certain directional hypotheses were theory-driven, the specific model configurations and effect patterns should be considered exploratory findings requiring replication.

A series of structural equation models were run to: (1) test direct effects of language ability on the four well-being outcomes after controlling for SES and PIQ, (2) test for moderation of these effects by SES, and (3) test for mediated indirect effects of language on SWL (hedonic well-being) by psychological well-being variables found to be directly associated with language. In all these models the measurement model was that described in Figure 2. Furthermore, the model fitting was the same as that used in the CFA implemented within the lavaan package of R (Rosseel, 2012). Standardized regression coefficients from the structural models were interpreted using conventional effect size guidelines, where coefficients of.10, .30, and.50 indicate small, medium, and large effects, respectively.

*Direct effects SEM model*. This model tested language regressions on the four well-being latent variables (satisfaction with life, scholastic ability, friends, and self-esteem). Additionally, we regressed the well-being latent variables on SES status, and PIQ to control for these variables.

*Moderation model*. This model tested the moderation of the association of language with each of the four well-being outcomes by SES. Separate models were run to simplify the models due to the additional complexity introduced by the interaction terms. in this case with an interaction term of SES by language serving as a moderator of the association between language and the well-being outcomes. Latent variable interactions were modeled using the product-indicator approach, which creates interaction indicators by multiplying each indicator of the language factor with each indicator of the SES factor (Marsh et al., 2004). This was implemented using the semTools package in R (Schoemann & Jorgensen, 2021). Therefore, the structural model included five regressions, in which the latent variables friends, scholastic ability, self-esteem, and SWL were regressed on language, PIQ, SES, and the language × SES interaction. To reduce nonessential multicollinearity between main effects and interaction terms, all indicators were double-centered (mean-centered within each pair) before creating the product indicators. Following Schoemann and Jorgensen's (2021) recommendations, we included the double-centered interaction error covariances due to our use of all-pairs missingness.

*Mediation SEM*. We tested a mediation SEM which had indirect paths from Language ability (conditioned on PIQ and SES) to SWL via Psychological Well-being variables found to be significantly associated with language ability in the direct effects model and thus potential mediators of language with SWL will be included in this model. The indirect pathway will provide a test of mediation. This model also contained a direct path between language ability and SWL. For the mediation model, indirect effects were calculated using bias-corrected bootstrap confidence intervals (*n* = 5,000 bootstrap samples) to test for significant mediation pathways.

*Power analyses*. We subscribe to the views of Amrhein et al. (2017) that *p*-values are a graded form of evidence against the null hypothesis that needs to be coupled with effect sizes and interval estimates. Thus, we adopted a *p* level of < .05 but considered values near this cutoff when the effect size was moderate or large. To ensure adequate statistical power for detecting meaningful effects, post-hoc power analyses were conducted for all SEM models. For the simple direct effects model (*N* = 502, df = 278) and mediation model (*N* = 502, df = 278), power exceeded 0.99 for detecting both medium (*β* ≥ 0.30) and small (*β* ≥ 0.20) effect sizes. For the more complex moderation model (*N* = 420, df = 618), power remained excellent for medium effects (> 0.99) and high for small effects (0.98). Model-level analyses indicated excellent power (>0.99) for detecting meaningful model misfit (RMSEA ≥ 0.05) across all models. These results suggest that nonsignificant effects likely reflect true absence of meaningful associations rather than insufficient statistical power.

## Results

### Direct effects of language on well-being

We investigated whether children's language abilities during the school years predicted adolescents’ self-reported sense of well-being after controlling for SES status, and PIQ. We tested this question by expanding the measurement model described above to include a structural model. The structural model added language regressions on the four well-being latent variables (SWL, scholastic ability, friends, and self-esteem). Additionally, we regressed the well-being latent variables on SES status, and PIQ to control for these variables. The statistical analysis used the same methods described for testing the fit and estimating parameters. This model was well fit to the data (χ²[278, *N* = 502] = 468.469, *p* = <.001; CFI = 0.941; TLI = 0.931; RMSEA = 0.037; SRMR = 0.051).

**Figure 3:**
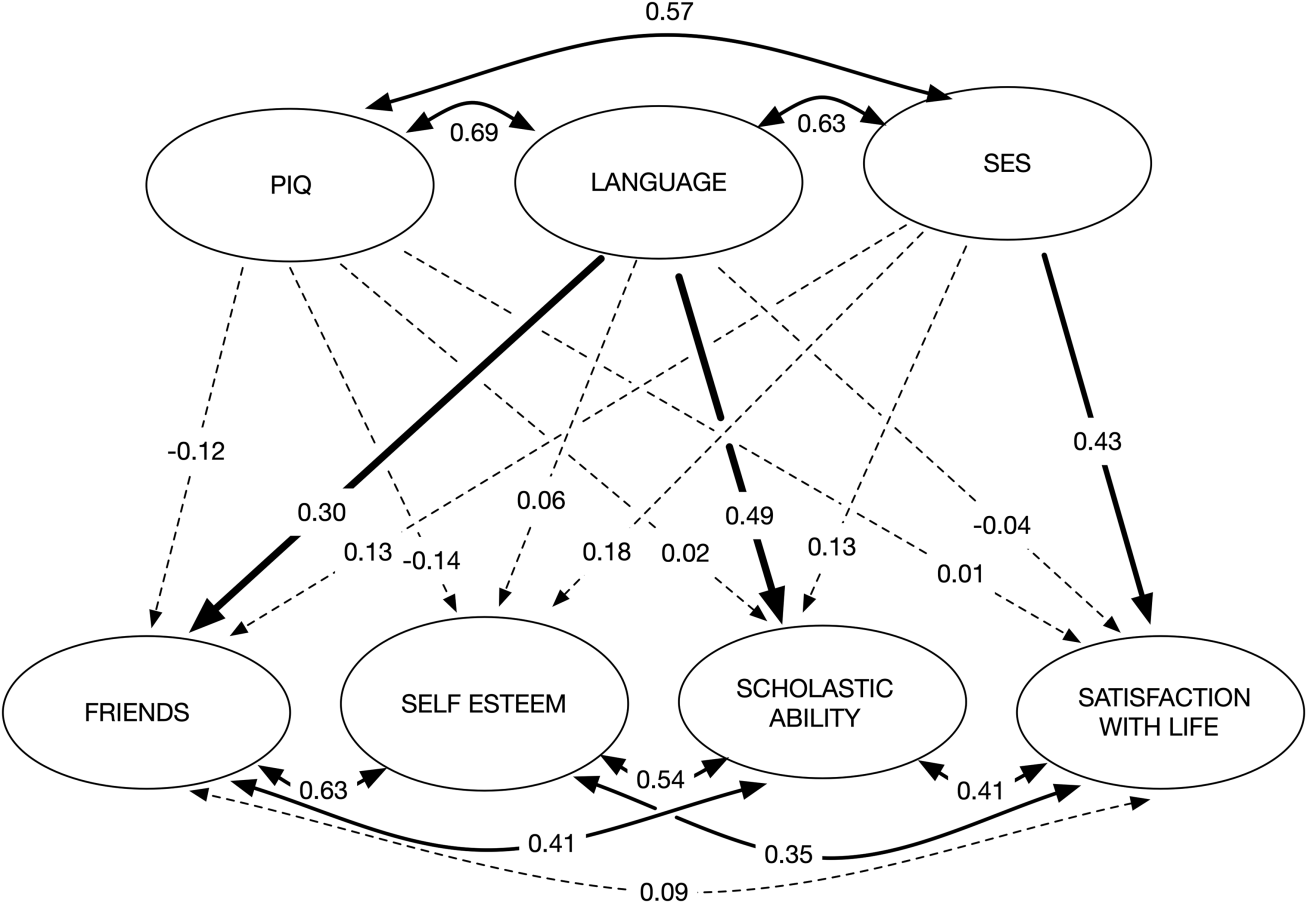
Model of language effects on well-being while controlling for performance IQ and parental SES. Significant (*p* < .001) standardized structural paths are shown in solid lines and nonsignificant paths are dashed lines. Language ability significantly predicted scholastic ability (*β* = 0.49) and friendships (*β* = 0.30) but not self-esteem or satisfaction with life.

Figure 3 provides the results showing the regressions and covariances of the latent variables in the structural model. These results showed significant positive associations of language ability with scholastic ability and social acceptance after controlling for PIQ and SES status. Thus, individual differences in language ability predicted scholastic ability and friends among adolescents with similar PIQ and SES status. The standardized regression coefficient of 0.49 for the association between language and scholastic ability means that for an increment or decrement of 1 *SD* in language is associated with a change of 0.49 *SD* in scholastic ability and 0.30 for friends. Thus, adolescents with similar performance IQs and SES backgrounds are likely to view themselves more positively in these two areas of well-being as a function of gains in their language ability. In contrast, language had a small nonsignificant association with self-esteem and SWL. SES was also found to be independently associated with SWL (*β*=0.43). Thus, adolescents from homes with better-educated parents who were earning more had higher hedonic levels of well-being and viewed themselves as doing better in school.

### Moderation of association between language ability and well-being by SES

In the primary analyses, SES was treated as a potential confounder in the association between Language ability and psychological well-being. However, SES may also serve as a moderator of this association. We initially constructed the interaction term based on 12 product indicators (4 language indicators and 3 SES indicators. These models had poor fit properties. Therefore, we simplified the interaction term to four product indicators (langk × med, lang2×fed, lang4×hincome, lang8×med). These models were fit to the data well: χ² (377, *N* = 502) = 579.04, *p* = <.001; CFI = 0.952; TLI = 0.944; RMSEA = 0.036; SRMR = 0.055.

The regression results and covariances of these structural models are detailed in Appendix F. None of the tests of the SES moderation of the association between language ability and the well-being outcomes were found to be significant by conventional standards. However, the language ability × SES interaction on scholastic ability (*β* = 0.242, *p* = .119) warrants more consideration, particularly due to the moderate effect size despite the statistical power demands of an interaction. Thus, we proceeded to explore this pattern using a Johnson-Neyman analysis that identifies the region of SES values where language effects on scholastic ability are significant (*p* < .05).

**Figure 4:**
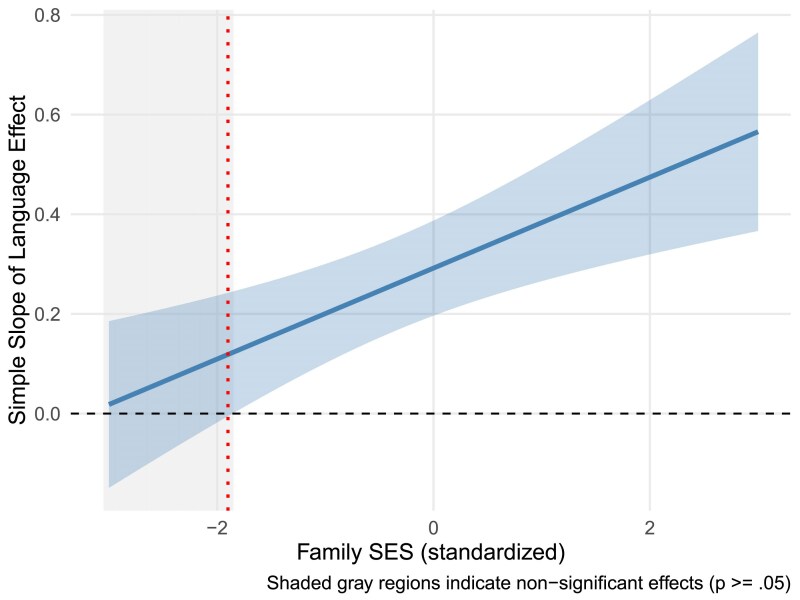
Johnson-Neyman plot of moderation of SES on language effects on scholastic ability.

As shown in Figure 4, the effect of language on the student's perceived scholastic ability becomes nonsignificant only for individuals with very low SES—those with SES levels below −1.8 standard deviations (bottom 6.2% of the sample). For most participants (93.8%), gains in language ability have a statistically significant positive effect on scholastic ability. This demonstrates that while there is a statistically nonsignificant language × SES interaction, the language effect remains significant across most of the SES distribution, becoming nonsignificant only at extremely low SES levels. This analysis supports the notion that the language effects on perceived scholastic ability are stronger in children from higher than lower SES homes.

### Mediation of language effects on SWL by scholastic ability and social acceptance

**Figure 5:**
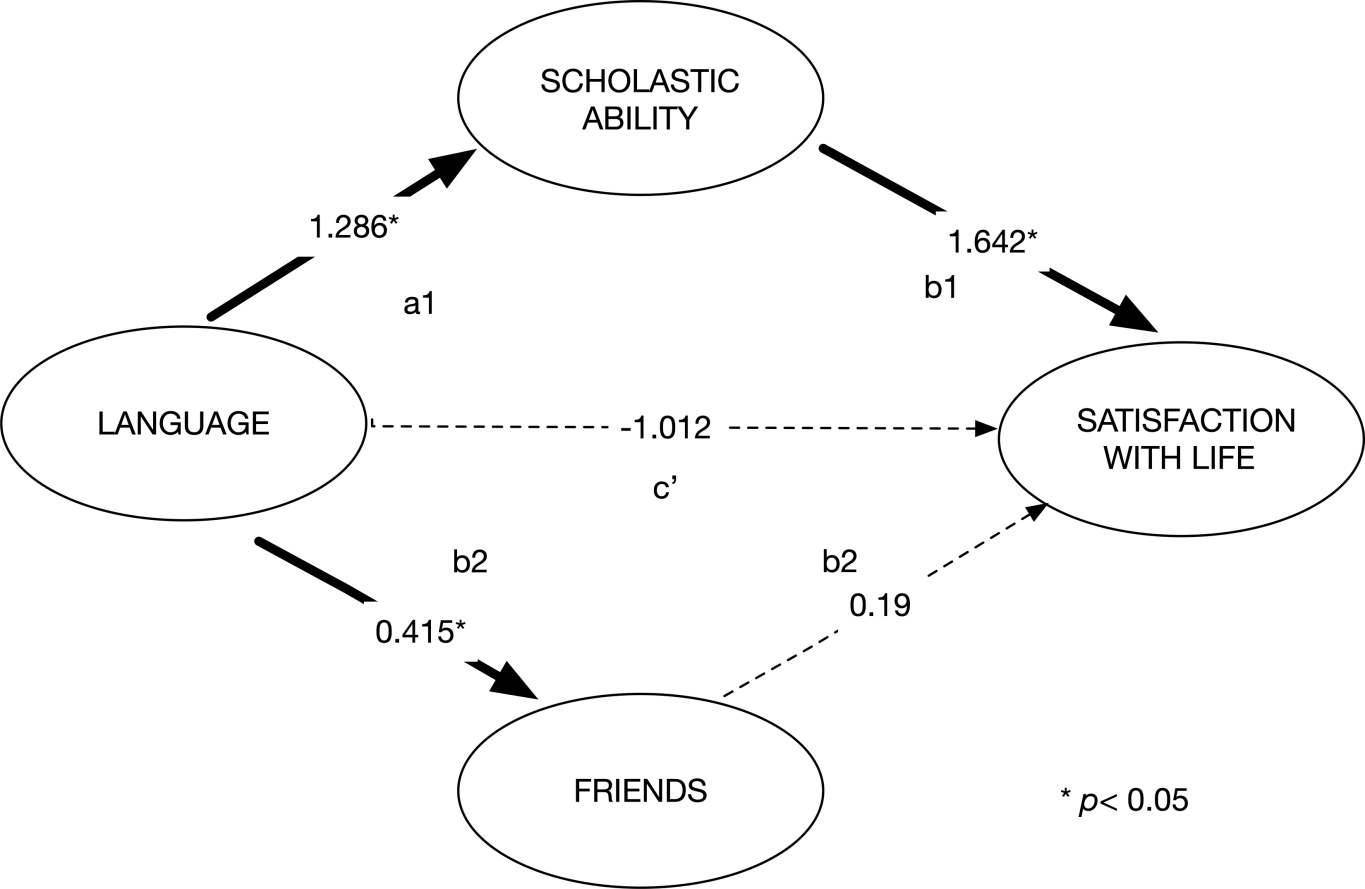
Mediation by scholastic ability and friends of effects of language on satisfaction with life.

The prior analyses showed that language ability predicts perceived scholastic ability and that this association is moderated by SES Status. However, there is no direct association of language ability with SWL. Given that SWL is reflective of hedonic well-being, we might expect that adolescents who are doing well scholastically and socially would be more satisfied with life. As noted in the introduction, prior work suggests that eudaimonic well-being such as scholastic performance or having friends might influence SWL and thus in this case function as a mediator of the association between language ability and SWL. The fact that our SWL measure was obtained a year after the psychological well-being measures allows us to examine for a predictive relationship between the eudaimonic and hedonic measures of well-being. This was tested via a mediation SEM where the structural model tested whether either scholastic ability or friendship quality mediate the relationship between language ability and SWL, while controlling for PIQ and SES. As shown in Figure 5, the models specified paths from language ability to either scholastic ability (a1) or friendship (a2) and from scholastic ability (b1) or friendship (b2) to SWL, and a direct path from language ability to SWL (c′), with the indirect effect calculated as the product of the a1 and b1 paths. The mediation models were estimated using both WLSMV and DWLS estimators. DWLS provided more precise parameter estimates and was selected for final interpretation based on superior standard error estimation for indirect effects.

We found that this model was well fit to the data: CFI = 0.948; TLI = 0.938; RMSEA = 0.046, 90%, SRMR = 0.056. Appendix G provides the regression results of the structural model along with the tests of the indirect paths. Figure 5 shows the loadings of the component paths. The total indirect effect was marginally significant *(β* = 0.845, 95% CI [−0.025, 1.715], *p* = .057). Decomposition revealed that this was driven by the scholastic ability pathway (*β* = 0.533, 95% CI [0.115, 0.952], *p* = .013), while the friendship pathway was not significant (*β* = 0.312, 95% CI [−0.342, 0.965], *p* = .350. The direct effect approached, but did not reach significance (*β* = −1.012, 95% CI [−2.238, 0.213], *p* = .106). The total effect (direct and indirect) of language ability on SWL was small and negative (*β* = −0.167, *p* > .05), indicating that the positive indirect effects through mediators were offset by the negative direct effect.

Although the direct effect just failed to reach the threshold of significance, the confidence interval shows a clear tendency toward a negative coefficient, which supported our further examination of the suppression effect which is consistent with our approach of treating *p*-values as graded evidence rather than strict thresholds. This pattern of opposing effects along the indirect (positive) and direct effects (negative) has been described as a suppression effect (Conger, 1974; MacKinnon et al., 2000). Following Muniz and MacKinnon (2025), we tested for statistical suppression using the criterion a1 × b1 × c′ < 0, where (see Figure 4) a1 is the language-scholastic ability coefficient, b1 is the scholastic ability-SWL coefficient, and c′ is the direct effect from language to SWL path. The calculation yielded a product of −0.534, supporting the presence of statistical suppression. The evidence of a suppression effect is likely to explain why we did not find a simple bivariate effect of language on SWL. The indirect path captures the variance in individual differences in language ability that is associated with scholastic accomplishment. This variance is in turn positively predictive of SWL. The direct path represents language variance that is unassociated with scholastic accomplishment and is weakly negatively predictive of subsequent SWL. These opposing processes result in a near zero effect of language on SWL masking the positive benefits that language ability confers through academic competence.

## Discussion

This study asked if individual differences in oral language abilities during the elementary school years influence personal well-being during later adolescence. The central findings of this study showed that the association between language and personal well-being was concentrated in the psychological well-being (eudaimonic) domains concerned with the adolescents’ perception of their scholastic performance and their friendship, but not their self-esteem. Language ability did not directly predict SWL; however, language ability was positively associated with SWL indirectly through scholastic ability. The SES status of the child's home was also found to both directly predict SWL and to moderate the association between language and scholastic ability, but not their perception of their friendships. We did not find an association of language with self-esteem; however, it along with scholastic ability was associated with SWL in the following year. Thus, these eudaimonic forms of well-being support the notion that they can predict and perhaps influence subsequent hedonic well-being as suggested by Keyes et al. (2002). In this regard, we should emphasize that the study design was longitudinal, and all the predictor variables were obtained a year or more before the outcome measures. Thus, the significant associations support, but do not confirm causal inferences.

### Effect of language on scholastic ability and SWL

The most robust relationship between language and well-being in general centered on the link between earlier language ability and scholastic ability. This relationship was found in all the models, and it was central to both the moderation and mediation models. It is not at all surprising that individual differences in language abilities were associated with the perception of scholastic competence. There is considerable evidence that children with poor language abilities tend to show lower levels of academic achievement, whether measured by teacher-assigned grades or standardized tests (Johnson et al., 1999; Stothard et al., 1998). Also, poorer academic outcomes are associated with depressed oral language ability due to variation in access to speech in children with hearing loss (Tomblin et al., 2020). Likewise, a large body of literature has demonstrated that educational outcomes among children being educated in their second language are associated with their proficiency in the second language (Genesee et al., 2005; Slama, 2012). Thus, school performance is strongly linked to oral language abilities regardless of the underlying reasons for language challenges. There is also a large body of literature examining the association between objective measures of academic performance and measures of personal well-being, often in the form of SWL (Kaya & Erdem, 2021; Steinmayr et al., 2016). Although the present study did not focus on the academic performance of these participants during the school years, these data were collected. A supplementary analysis revealed that teacher-reported academic performance in second and fourth grades was significantly associated with the adolescents’ self-perceived scholastic ability (*β* = 0.29). Therefore, we can conclude that objective measures of language are associated with personal judgments of academic competence, and this association is mediated by actual academic performance. Through this pathway, children may construct a sense of their cognitive and intellectual abilities, which becomes a dimension of their sense of psychological well-being.

The moderation of the language-scholastic ability relationship by SES can be explained by considerable evidence that SES is strongly associated with cultural values that are concerned with academic performance (Lareau, 2003). In this study, SES is based on parental education and income. Thus, SES is strongly reflective of parental attitudes regarding educational achievement and therefore the children in this study were likely to be enculturated with this value system. As a result, the strength of the language-scholastic ability association varies according to SES level, reflecting the cultural values of the home. This finding demonstrates that the cultural valuation of academic achievement within higher-SES families amplifies the relationship between language ability and perceived scholastic competence, consistent with Bourdieu's theory of cultural capital reproduction (Bourdieu, 1991; Sullivan, 2001).

We performed a mediation analysis to test whether the association between language ability and SWL might act through scholastic ability This analysis allows for both a direct association between language ability and SWL and a mediated indirect association. This was motivated by prior research and theory regarding the interplay between eudaimonic and hedonic aspects of well-being. This analysis revealed a negative direct association between language ability and SWL and a positive indirect association between language ability and SWL that is mediated by scholastic ability afforded by language. The strength of this mediated effect was 0.626 (standardized *β* = 0.533), which represents a large indirect effect. Thus, as lesser or greater language ability translates into lesser or greater perceived academic competence, we see these effects translate into the hedonic measure of SWL. This mediation process depends on a positive association between language and academic performance during the school years that may subsequently influence the eudaimonic perception of academic competence which in turn may influence SWL. These findings provide support for the notion that eudaimonic well-being can influence hedonic well-being as hypothesized by Keyes et al. (2002) and shown by (Joshanloo, 2019) and aligns with self-determination theory, which argues that psychological need satisfaction—particularly competence—supports both optimal functioning and subjective well-being (DeHaan & Ryan, 2014).

The negative direct association between language ability and SWL was not expected. This pattern of negative and positive associations along the direct and indirect paths has been found in other research and is known as a suppression effect (Conger, 1974). The presence of this negative association helps explain why we did not find a significant association between language ability and SWL in the initial (unmediated) model—the negative direct path counters the positive effects of the indirect path. We can only speculate what the basis of this negative association is. Within the model, the indirect association absorbs the variation in language ability that is independent of scholastic ability. This leaves the residual variance in language that is unrelated to scholastic ability to be associated directly with SWL. This residual variance may reflect aspects of language ability that create elevated academic expectations. When these expectations are not fully realized through scholastic achievement, they may contribute to lower life satisfaction—a pattern consistent with research on academic underachievement and psychological distress (Blaas, 2014).

### Effect of language on perception of friendships

We also found that individual differences in language ability predicted the reports of the adolescents having close friends, not feeling lonely and feeling comfortable talking. This relationship is consistent with the fact that a principal function of language is the construction and maintenance of interpersonal relationships. These data are consistent with empirical studies that have shown effects of language on children's friendships (Chow et al., 2022; Durkin & Conti-Ramsden, 2007). Importantly, unlike scholastic ability, the language-friendship relationship was not moderated by SES, suggesting that social benefits of language ability may be more universal across socioeconomic contexts. Huta and Waterman (2013) showed that several researchers considered relatedness, positive relationships and social well-being were features of eudaimonic forms of well-being. Thus, these data show that language abilities are likely to contribute to eudaimonic well-being.

### Strengths and limitations

This study drew on data from a substantial 10-year longitudinal investigation children, originally selected from a population sample of nearly 7,000 kindergarteners. This large-scale, population-based design provides several methodological strengths. First, the longitudinal nature of the study, with language measures collected across four time points during elementary school years and well-being outcomes assessed in adolescence, strengthens the basis for causal inferences about language effects on later well-being. Also, the large sample size provided adequate statistical power for detecting meaningful effects in complex structural equation models, including moderation and mediation analyses. While the data reflect cultural values of Midwest U.S. adolescents in 2005, the fundamental relationships between language ability and well-being likely transcend specific generational effects, though replication across different cultural contexts and time periods would strengthen these conclusions. This study operationalized the construct of language abilities to be those involved in the comprehension and production of utterances (words, sentences and connected text) within a standardized testing context. We did not incorporate language functions that involve more complex social-pragmatic functions. In this respect the findings of this study may underestimate the influence of broader communication skills on well-being. It is noteworthy that some of the measures of well-being, particularly psychological well-being, in this study did not have the benefit of the research and theory development that has been conducted over the past 20 years and thus are not ideal. The measures of psychological well-being used here were based on the *Perceived Competence Scale* and have not been explicitly acknowledged as measures of psychological well-being in contrast with the *Psychological Well-Being scales* developed by Ryff and Keyes (1995). In this regard, this study does not provide a comprehensive examination of language ability and psychological well-being.

## Conclusion

This study was aimed at examining whether individual differences in oral language ability during the elementary grades are associated with subsequent self-reported indicators of well-being in adolescence. The findings support the notion that individual differences in oral language ability participate in a complex developmental network involving nonlinear interactions among home and school to affect the construction of an adolescent's personal perspective of academic and social self-competence and worth and subsequently SWL. These findings have important implications for educational interventions, suggesting that supporting language development may enhance well-being through both academic and social pathways.

## Supplementary Material

aacaf057_Supplementary_Data

## Data Availability

The data and analytic code necessary to reproduce the analyses presented here are openly available at the Open Science Framework (https://osf.io/https://osf.io/4hwa6) under the project title “Language and Well-being.”
