# Supplementary material for: Individual differences in language during childhood predict well-being in adolescence
Source: Child Dev. 2026 Feb 26;97(2):352–65. doi: 10.1093/chidev/aacaf057 (PMC13046077; doi:10.1093/chidev/aacaf057)
Supplement: aacaf057_Supplementary_Data [file aacaf057_supplementary_data.docx]

Appendices

| ***Appendix A****.*  **Table A1**  ***Measures of Spoken Language Obtained across the Longitudinal Study.*** | | | | |
| --- | --- | --- | --- | --- |
| Year | Modality | Word | Sentence | Discourse |
| Kindergarten | Rec | TOLD: Picture ID | TOLD: Grammatic Understanding | Culatta: Narrative Comprehension |
|  | Exp | TOLD: Oral Vocabulary | TOLD: Grammatic Completion & Sentence Imitation | Cullatta: Narrative Recall |
| 2 | Rec | PPVT-R | CELF-III  Sentence Structure  Concepts & Direct. | CELF-III  List. to Paragraphs |
|  | Exp | CREVT-Expressive | CELF-III  Recalling Sentences  Word Structure | Fey/Catts:  Story Generation |
| 4 | Rec | PPVT-R | CELF-III  Concepts & Direct. | CELF-III  List. to Paragraphs |
|  | Exp | CREVT-Expressive | CELF-III  Recalling Sentences  Formulated Sentences | Fey/Catts:  Story Generation |
| 8 | Rec | PPVT-R | CELF-III  Concepts & Direct. | Expository Listening Comprehension Recall |
|  | Exp | CREVT-Expressive | CELF-III  Recalling Sentences | Expository Recall |
| TOLD:2P Test of Oral Language Development  CELF-III: Clinical Evaluation of Language Fundamentals (3^rd^ ed.)  CREVT: Comprehensive Receptive & Expressive Vocabulary Test  GORT-III: Gray Oral Reading Tests (3^rd^ ed.) | | | | |

| ***Appendix B****.*  **Table B1**  ***Items used in Psychological Well-being Scale.*** | | | | | |
| --- | --- | --- | --- | --- | --- |
| Variable | Left side |  | | Right Side | |
| Satisfy with self | Some students are often dissatisfied with themselves |  | | Other students are usually satisfied with themselves. | |
| Like self | Some students like the kind of person they are. | |  | Other students wish they were different. |  |
| Not lonely | Some students get kind of lonely because they don’t have a close friend to share things with. |  | | Other students don’t usually get too lonely because they do have a close friend to share things with. | |
| Close friends | Some students have a friend who is close enough for them to share thoughts that are personal |  | | Other students don’t have a close friend they can share their personal thoughts and feelings with | |
| Creative | Some students think that they are not as creative or inventive as other people. |  | | Other students feel they are very creative and inventive. | |
| Do well in school | Some students don’t do very well in their schoolwork. |  | | Other students do very well with their schoolwork. | |
| Confident in school | Some students feel confident they are mastering their schoolwork |  | | Other students do not feel so confident that they are mastering their schoolwork. | |
| Understand teacher | Some students have trouble understanding what the teacher says. |  | | Other students find it easy to understand what the teacher says. | |
| Get along with parents | Some students find they are unable to get along with their parents. |  | | Other students get along with their parents quite well. | |
| Smart | Some students feel like they are just as smart or smarter than other students |  | | Other students wonder they are as smart. | |
| Bright | Some students wonder if they are as bright as most people |  | | Other students feel they are just as bright or brighter than most people. | |
| Good at sports | Some students feel they are better than others at sports |  | | Other students don’t feel they can play well. | |
| Romantic relations | Some students have the ability to develop romantic relationships. |  | | Other students do not find it easy to develop romantic relationships. | |
| Like appearance | Some students like their physical appearance the way it is |  | | Other students do not like their physical appearance. | |
| Comfortable talking | Some students are comfortable in situations where they have to talk. |  | | Other students are not comfortable in situation where they have to talk. | |
| Good dancer | Some students think they are good dancers |  | | Other student think they aren’t good at dancing | |

***Appendix C***

| **Table C1.**  ***Descriptive Statistics of Indicator Variables (weighted).*** | | | | | |
| --- | --- | --- | --- | --- | --- |
| Variable | Mean | Median | SD | Skewness | Kurtosis |
| piqk | 98.59 | 100.00 | 14.93 | -0.15 | 0.40 |
| piq2 | 100.53 | 100.00 | 15.06 | -0.07 | -0.16 |
| piq8 | 100.51 | 100.00 | 15.94 | 0.05 | 0.70 |
| langk | 0.06 | 0.19 | 1.08 | -0.10 | -0.44 |
| lang2 | 0.03 | 0.07 | 1.00 | -0.28 | 0.06 |
| lang4 | 0.03 | 0.05 | 1.01 | -0.38 | 1.11 |
| lang8 | 0.04 | 0.09 | 0.99 | -0.38 | 0.17 |
| not_lonely | 3.42 | 4.00 | 0.81 | -1.24 | 0.64 |
| close_friend | 3.57 | 4.00 | 0.71 | -1.66 | 2.24 |
| parents | 2.97 | 3.00 | 0.95 | -0.52 | -0.74 |
| do_well | 2.85 | 3.00 | 0.91 | -0.37 | -0.69 |
| confident | 2.77 | 3.00 | 0.85 | -0.26 | -0.54 |
| smart | 2.90 | 3.00 | 0.88 | -0.39 | -0.61 |
| creative | 2.67 | 3.00 | 0.86 | -0.14 | -0.65 |
| bright | 2.78 | 3.00 | 0.87 | -0.14 | -0.80 |
| comfortable | 2.93 | 3.00 | 0.93 | -0.48 | -0.67 |
| romantic | 2.89 | 3.00 | 0.88 | -0.37 | -0.65 |
| sports | 2.80 | 3.00 | 0.90 | -0.35 | -0.65 |
| appearance | 2.92 | 3.00 | 0.86 | -0.38 | -0.60 |
| understanding | 2.88 | 3.00 | 0.89 | -0.34 | -0.71 |
| dance | 2.41 | 2.00 | 1.05 | 0.06 | -1.22 |
| like_self | 3.26 | 3.00 | 0.80 | -0.86 | 0.09 |
| satisfy | 2.95 | 3.00 | 0.86 | -0.46 | -0.47 |
| life_1 | 5.13 | 6.00 | 1.36 | -1.05 | 0.55 |
| life_2 | 5.29 | 6.00 | 1.46 | -1.03 | 0.30 |
| life_3 | 5.63 | 6.00 | 1.41 | -1.46 | 1.85 |
| life_4 | 5.16 | 6.00 | 1.52 | -0.87 | 0.03 |
| fed | 13.71 | 13.00 | 2.54 | 0.66 | 0.48 |
| med | 13.98 | 14.00 | 2.26 | 0.58 | 0.29 |
| Household income | 3.52 | 4.00 | 1.57 | -0.56 | -0.16 |

piqk=performance IQ kindergarten, piq2=performance IQ second grade, piq8=performance IQ eighth grade, langk=language kindergarten, lang2=language second grade, lang4=language fourth grade, lang8=language eighth grade, fed=father’s education, med=mother’s education.

|  |
| --- |
|  |

**Appendix D**.

*Correlation among Indicator Variables*

| **Table D1**  ***Pearson correlations between Continuous Variables*** | | | | | | | | | |
| --- | --- | --- | --- | --- | --- | --- | --- | --- | --- |
| Variable | piqk | piq2 | piq8 | langk | lang2 | lang4 | lang8 | fed | med |
| piq2 | 0.71 |  |  |  |  |  |  |  |  |
| piq8 | 0.64 | 0.71 |  |  |  |  |  |  |  |
| langk | 0.46 | 0.57 | 0.47 |  |  |  |  |  |  |
| lang2 | 0.46 | 0.53 | 0.47 | 0.79 |  |  |  |  |  |
| lang4 | 0.45 | 0.54 | 0.52 | 0.77 | 0.85 |  |  |  |  |
| lang8 | 0.50 | 0.58 | 0.56 | 0.75 | 0.80 | 0.83 |  |  |  |
| fed | 0.34 | 0.35 | 0.34 | 0.41 | 0.37 | 0.41 | 0.47 |  |  |
| med | 0.31 | 0.33 | 0.27 | 0.41 | 0.36 | 0.40 | 0.44 | 0.60 |  |
| household income | 0.28 | 0.30 | 0.31 | 0.29 | 0.29 | 0.27 | 0.40 | 0.39 | 0.38 |
| piqk=performance IQ kindergarten, piq2=performance IQ second grade, piq8=performance IQ eighth grade, langk=language kindergarten, lang2=language second grade, lang4=language fourth grade, lang8=language eighth grade, fed=father’s education, med=mother’s education. | | | | | | | | | |

***Table D2***

***Spearman Correlations between Ordinal Variables***

***Table D3****.*

***Spearman Correlations between Ordinal and Continuous (Contn.)***

piqk=performance IQ kindergarten, piq2=performance IQ second grade, piq8=performance IQ eighth grade, langk=language kindergarten, lang2=language second grade, lang4=language fourth grade, lang8=language eighth grade, fed=father’s education, med=mother’s education. hinco=household income

***Appendix E.***

**Table E1**

***Standardized loadings, z-values, Composite Reliabilities and Average Variance Extracted From the 8***

***Factor Confirmatory Factor Analysis***

| Factor | Indicator Variable | Standardized  Estimate | Std.Err | z-value | Composite Relibility | Ave. Var. Extracted |
| --- | --- | --- | --- | --- | --- | --- |
| SELF ESTEEM |  |  |  |  | 0.722 | 0.396 |
|  | Like appearance | 0.59 | 0.048 | 12.19 |  |  |
|  | Positive parent relationship | 0.556 | 0.054 | 10.221 |  |  |
|  | Self-satisfied | 0.666 | 0.062 | 9.302 |  |  |
|  | Like self | 0.694 | 0.056 | 9.984 |  |  |
| SCHOLASTIC  ABILITY |  |  |  |  | 0.827 | 0.49 |
|  | Good school performance | 0.68 | 0.036 | 18.819 |  |  |
|  | Confident with school | 0.7 | 0.031 | 22.253 |  |  |
|  | Smart | 0.724 | 0.032 | 22.419 |  |  |
|  | Understand teacher | 0.769 | 0.031 | 24.624 |  |  |
|  | Bright | 0.619 | 0.042 | 14.852 |  |  |
| PHYSICAL  SOCIAL ABILITY |  |  |  |  | 0.53 | 0.285 |
|  | Have romantic relationship | 0.677 | 0.062 | 10.96 |  |  |
|  | Good dancer | 0.391 | 0.061 | 6.446 |  |  |
|  | Athletic | 0.493 | 0.06 | 8.286 |  |  |
| FRIENDS |  |  |  |  | 0.625 | 0.36 |
|  | Not lonely | 0.603 | 0.063 | 9.572 |  |  |
|  | Have close friend | 0.519 | 0.061 | 8.509 |  |  |
|  | Comfortable talking | 0.668 | 0.057 | 11.617 |  |  |
| SWL |  |  |  |  | 0.877 | 0.643 |
|  | life_1 | 0.799 | 0.026 | 31.229 |  |  |
|  | life_2 | 0.891 | 0.022 | 40.538 |  |  |
|  | life_3 | 0.849 | 0.025 | 34.56 |  |  |
|  | life_4 | 0.647 | 0.036 | 17.96 |  |  |
| LANGUAGE |  |  |  |  | 0.942 | 0.803 |
|  | Kindergarten language | 0.852 | 0.055 | 16.601 |  |  |
|  | Grade 2 language | 0.869 | 0.043 | 20.381 |  |  |
|  | Grade 4 language | 0.914 | 0.033 | 27.773 |  |  |
|  | Grade 8 language | 0.947 | 0.039 | 24.248 |  |  |
| SES |  |  |  |  | 0.725 | 0.469 |
|  | Mother's education | 0.715 | 0.126 | 12.793 |  |  |
|  | Father's education | 0.73 | 0.142 | 13.063 |  |  |
|  | Home income | 0.604 | 0.103 | 9.196 |  |  |
| PIQ |  |  |  |  | 0.872 | 0.694 |
|  | Kindergarten PIQ | 0.796 | 0.714 | 16.658 |  |  |
|  | Grade 2 Performance IQ | 0.876 | 0.75 | 17.589 |  |  |
|  | Grade 8 Performance IQ | 0.824 | 0.675 | 19.475 |  |  |

All loading values have *p* <0.0001

| \| Appendix F.  Table F1.  Regression Results Across Four Modertion SEM Models \| \| \| \| \| \| \| \| --- \| --- \| --- \| --- \| --- \| --- \| --- \| \| Outcome \| Predictor \| Estimate \| SE \| z_value \| p_value \| Sandardized Estimate \| \| FRIENDS \|  \|  \|  \|  \|  \|  \| \|  \| LANGUAGE \| 0.371 \| 0.115 \| 3.238 \| 0.001 \| 0.345 \| \| PIQ \| -0.131 \| 0.151 \| -0.864 \| 0.388 \| -0.122 \| \| SES \| 0.14 \| 0.331 \| 0.423 \| 0.673 \| 0.13 \| \| LANGUAGE X SES interaction \| 0.002 \| 0.275 \| 0.008 \| 0.994 \| 0.002 \| \| SCHOLASTIC ABILITY \|  \|  \|  \|  \|  \|  \| \|  \| LANGUAGE \| 0.538 \| 0.082 \| 6.592 \| 0 \| 0.428 \| \| PIQ \| 0.133 \| 0.107 \| 1.234 \| 0.217 \| 0.106 \| \| SES \| -0.087 \| 0.241 \| -0.361 \| 0.718 \| -0.069 \| \| LANGUAGE X SES interaction \| 0.304 \| 0.195 \| 1.561 \| 0.119 \| 0.242 \| \| ESTEEM \|  \|  \|  \|  \|  \|  \| \|  \| LANGUAGE \| 0.037 \| 0.073 \| 0.5 \| 0.617 \| 0.036 \| \| PIQ \| -0.115 \| 0.106 \| -1.081 \| 0.28 \| -0.113 \| \| SES \| 0.273 \| 0.244 \| 1.115 \| 0.265 \| 0.268 \| \| LANGUAGE X SES interaction \| -0.092 \| 0.201 \| -0.46 \| 0.645 \| -0.091 \| \| SWL \|  \|  \|  \|  \|  \|  \| \|  \| LANGUAGE \| -0.047 \| 0.086 \| -0.55 \| 0.582 \| -0.043 \| \| PIQ \| -0.045 \| 0.134 \| -0.339 \| 0.735 \| -0.041 \| \| SES \| 0.71 \| 0.349 \| 2.036 \| 0.042 \| 0.642 \| \| LANGUAGE X SES interaction \| -0.246 \| 0.275 \| -0.895 \| 0.371 \| -0.223 \| \|  \| \| \| \| \| \| \| |
| --- | --- | --- | --- | --- | --- | --- | --- | --- | --- | --- | --- | --- | --- | --- | --- | --- | --- | --- | --- | --- | --- | --- | --- | --- | --- | --- | --- | --- | --- | --- | --- | --- | --- | --- | --- | --- | --- | --- | --- | --- | --- | --- | --- | --- | --- | --- | --- | --- | --- | --- | --- | --- | --- | --- | --- | --- | --- | --- | --- | --- | --- | --- | --- | --- | --- | --- | --- | --- | --- | --- | --- | --- | --- | --- | --- | --- | --- | --- | --- | --- | --- | --- | --- | --- | --- | --- | --- | --- | --- | --- | --- | --- | --- | --- | --- | --- | --- | --- | --- | --- | --- | --- | --- | --- | --- | --- | --- | --- | --- | --- | --- | --- | --- | --- | --- | --- | --- | --- | --- | --- | --- | --- | --- | --- | --- | --- | --- | --- | --- | --- | --- | --- | --- | --- | --- | --- | --- | --- | --- | --- | --- | --- | --- | --- | --- | --- | --- | --- | --- |

| **Table F2**  ***Covariances of Moderation SEM*** | | | | | | | | | | |  |
| --- | --- | --- | --- | --- | --- | --- | --- | --- | --- | --- | --- |
| Covariates | | Estimate | | | | Std.Err | | z-value | | P(>\|z\|) |  |
| LANGUAGE |  | | |  | | |  | |  |  |  |
|  | SES | | 0.627 | | 0.037 | | | | 17.014 | 0 |  |
|  | PIQ | | 0.687 | | 0.031 | | | | 22.088 | 0 |  |
|  | LANGUAGE x SES_ | | 0.5 | | 0.019 | | | | 26.002 | 0 |  |
| SES |  | |  | |  | | | |  |  |  |
|  | PIQ | | 0.548 | | 0.042 | | | | 13.031 | 0 |  |
|  | LANGUAGE x SES | | 0.829 | | 0.047 | | | | 17.624 | 0 |  |
| PIQ |  | |  | |  | | | |  |  |  |
|  | LANGUAGE x SES | | 0.301 | | 0.024 | | | | 12.294 | 0 |  |
| .ESTEEM |  | |  | |  | | | |  |  |  |
|  | .FRIENDS | | 0.622 | | 0.064 | | | | 9.722 | 0 |  |
|  | .SWL | | 0.38 | | 0.039 | | | | 9.667 | 0 |  |
|  | .SCHOLASTIC_ABILITY | | 0.543 | | 0.047 | | | | 11.658 | 0 |  |
| .FRIENDS |  | |  | |  | | | |  |  |  |
|  | .SWL | | 0.072 | | 0.065 | | | | 1.112 | 0.266 |  |
|  | .SCHOLASTIC_ABILITY | | 0.464 | | 0.065 | | | | 7.089 | 0 |  |
| .SWL |  | |  | |  | | | |  |  |  |
|  | .SCHOLASTIC_ABILITY | | 0.264 | | 0.052 | | | | 5.085 | 0 |  |

**Appendix G**.

**Table G1**

***Results of Structural Equation Modeling of Mediating Effects of Scholastic Ability and Friends on the Relationship between Language and Satisfaction with Life with Performance IQ and Socioeconomic Status functioning as confounders***

| Regressions: |  |  |  |  |  |  | |  | |  |
| --- | --- | --- | --- | --- | --- | --- | --- | --- | --- | --- |
|  | Estimate | SE | z-value | P(>\|z\|) | Standardized  Coefficient | |  |  |  |  |
| SCHOLASTIC ABILITY |  |  |  |  |  |  | | |  |  |
|  | LANG (a1) | 0.408 | 4.586 | 0 | 1.286 |  | | |  |  |
|  | PIQ | 0.345 | -3.207 | 0.001 | -0.759 |  | | |  |  |
|  | SES | 0.159 | 0.532 | 0.595 | 0.058 |  | | |  |  |
| FRIENDS |  |  |  |  |  |  | | |  |  |
|  | LANG (a2) | 0.815 | 2.98 | 0.003 | 1.642 |  | | |  |  |
|  | PIQ | 0.726 | -2.725 | 0.006 | -1.339 |  | | |  |  |
|  | SES | 0.246 | -0.017 | 0.986 | -0.003 |  | | |  |  |
| SWL |  |  |  |  |  |  | | |  |  |
|  | SCHOLAS (b1) | 0.101 | 3.3 | 0.001 | 0.415 |  | | |  |  |
|  | FRIENDS (b2) | 0.141 | 1.067 | 0.286 | 0.19 |  | | |  |  |
|  | LANG (c') | 0.792 | -1.499 | 0.134 | -1.012 |  | | |  |  |
|  | PIQ | 0.62 | 1.276 | 0.202 | 0.674 |  | | |  |  |
|  | SES | 0.156 | 3.289 | 0.001 | 0.438 |  | | |  |  |
| Indirect Scholastic Ability |  | 0.274 | 2.283 | 0.001 | 0.533 |  | | |  | |
| Indirect Friends |  | 0.405 | 0.903 | 0.001 | 0.312 |  | | |  | |
| total indirect |  | 0.566 | 1.751 | 0.001 | 0.845 |  | | |  | |

| Covariances: |  |  |  |  |  |  |
| --- | --- | --- | --- | --- | --- | --- |
|  |  | Estimate | SE | z-value | P(>\|z\|) | Standardized  Coefficient |
| LANG |  |  |  |  |  |  |
|  | SES | 0.668 | 0.039 | 16.946 | 0 | 0.668 |
|  | PIQ | 0.897 | 0.025 | 36.365 | 0 | 0.897 |
| SES |  |  |  |  |  |  |
|  | PIQ | 0.604 | 0.046 | 12.995 | 0 | 0.604 |
